# Impact of aging on obstetric outcomes: defining advanced maternal age in Barcelona

**DOI:** 10.1186/s12884-019-2415-3

**Published:** 2019-09-23

**Authors:** Marta Claramonte Nieto, Eva Meler Barrabes, Sandra Garcia Martínez, Mireia Gutiérrez Prat, Bernat Serra Zantop

**Affiliations:** Gran Via de Carles III, 71-75, 08028 Barcelona, Spain

**Keywords:** Advanced maternal age, Outcomes, Gestational diabetes, Preeclampsia, C-section, Placenta previa, Prematurity

## Abstract

**Background:**

Women of advanced maternal age (AMA) are a growing population, with higher obstetric risks. The Mediterranean population has specific characteristics different from other areas. Thus, the objective of this study was to establish a cut-off to define AMA in a selected mediterranean population coming from a tertiary referral private/mutual health hospital in Barcelona.

**Methods:**

Retrospective cohort of euploid singleton pregnancies delivered from January 2007 to June 2017. Main maternal outcomes were: gestational diabetes, preeclampsia, placenta previa, c-section and prolonged hospitalization (≥ 7 days). Main adverse perinatal outcomes were: stillbirth, prematurity, preterm prelabor rupture of membranes, low birth weight, need of admission at a neonatal intensive care unit and perinatal mortality. Adjustment for confounding factors (smoking, previous comorbilities, parity, assisted reproductive techniques (ART) and obesity) was performed.

**Results:**

A total of 25054 pregnancies were included. Mean maternal age was 34.7 ± 4.2 years, with 2807 patients in the group of age between 40 and 44 years (11.2%) and 280 patients ≥45 years (1.1%). Women at AMA had higher incidence of previous comorbilities (compared to the reference group of women < 30 years): prior c-section, chronic hypertension and obesity. In addition, they were more likely to use ART. After adjusting for confounding factors, maternal age was an independent and statistically significant risk factor for gestational diabetes (OR 1.66/2.80/3.14) for ages 30–39, 40–44 and ≥ 45 years respectively, c-section (OR 1.28/2.41/7.27) and placenta previa (OR 2.56/4.83) for ages 40–44 and ≥ 45 years respectively, but not for preeclampsia (neither early-onset nor late-onset). Risk of emergency c-section was only increased in women ≥45 years (OR, 2.03 (95% CI, 1.50–2.74). In the other groups of age, the increase in c-section rate was because of elective indications. Age ≥ 45 years was associated with iatrogenic prematurity < 37 weeks (OR 2.62, 95% CI 1.30–5.27). No other relevant associations between AMA and maternal or neonatal outcomes were found.

**Conclusions:**

Maternal age is an independent risk factor for adverse obstetric outcomes. Age ≥ 40 years was associated to relevant increased risks and reveals to be an adequate cut-off to define AMA in our population.

## Background

In the last decades, the rate of pregnancies at an advanced maternal age (AMA) has increased steadily [1–3]. In most studies addressing this issue, AMA is defined as maternal age above 35 years, considering very advanced maternal age (VAMA) 40 years or more and extremely advanced maternal age (EAMA) 45 years or more [4].

In Spain, mean maternal age at first delivery has risen from 25.2 years in 1975 to 30.7 years in 2016. In 2016, 38.7% of deliveries in Spain were among mothers over 35 years of age and 8.39% over 40 [1]. The delay of motherhood is especially obvious in high-income countries [5, 6]. A higher educational level, a delayed economic independence, difficulties in conciliating and the widespread use of assisted reproductive techniques may explain this tendency [2].

Recent predictive demographic models in Spain pin mean maternal age of the first pregnancy around 33 years by the year 2050 [3]. Thus, the scientific community is concerned about the impact of AMA on obstetrical outcomes, and aims to continuously update knowledge about AMA and its attributable obstetric consequences, in order to better categorize and assess this increasing subgroup of pregnant women [4, 7–16] .

Most studies agree that AMA is related to an increase of adverse maternal and fetal outcomes such as preeclampsia (PE), gestational diabetes (GD), stillbirth, placenta previa (PP), intrauterine growth restriction (IUGR), prematurity and cesarean section. However, one of the main limitation of some published data is the suboptimal control of some important confounding factors such as maternal chronic hypertension, smoking status, or previous c-section [8]. Moreover, some other studies did not include multivariable logistic regression analysis in the results [12, 15] a major limitation as some outcomes reveal not to be significantly increased when controlling for confounding factors [7, 9].

Finally, and not less negligeable, most of the studies omit the subgroup > 45 years old because of a reduced number of patients, thus potentially underestimating the risk in that group of patients. However, latest studies suggest a cut-off to define AMA ≥40 years, based on risk prediction charts [17].

In that context, we aimed to accurately assess and validate the cut-off to define AMA in our Mediterranean population according to specific obstetrical outcomes. For that purpose, we conducted a study evaluating prior maternal comorbidities and the association of AMA with obstetrical outcomes.

## Methods

This was a retrospective cohort study conducted between January 2007 and June 2017 on women who attended antenatal visits and delivered in Salud de la Mujer Dexeus, a private/mutual health tertiary university hospital in Barcelona,.

All pregnancies included in the study were from singleton pregnant women over 18 years at the time of the delivery and who gave birth after 23 weeks of gestation. Pregnancies with aneuploidies or microdeletions confirmed by karyotype or array-CGH, major fetal defects and multiple gestations were excluded.

At the first antenatal visit, maternal characteristics were collected: previous maternal conditions (chronic hypertension, diabetes mellitus type I or II, thrombophilia, heart disease, renal disease, neurological disease, asthma and psychiatric disorders), cigarette smoking status and other medical conditions. We also recorded previous obstetric history: parity, previous c-section, history of previous preeclampsia, gestational diabetes, stillbirth or low birth weight. The method of conception (spontaneous vs. assisted reproductive techniques) was also registered. Maternal height (cm) and weight (kg) were measured in first visit. Database accuracy was validated by reviewing the information in 250 patients, manually checking that there were no systematic mistakes in data introduction.

Gestational age was determined by first trimester CRL ultrasound measurement [18]. When first trimester scan was not available, gestational age was determined by last menstruation period.

Adverse maternal outcomes considered were: preeclampsia, gestational diabetes, stillbirth, placenta previa, preterm prelabor rupture of membranes (PPROM) (defined below 37 weeks), chorioamnionitis and preterm delivery. Preterm deliveries were in turn divided in extreme preterm (below 28 weeks), moderate preterm (below 34 weeks) and late preterm (below 37 weeks). Cause of prematurity, whether spontaneous or iatrogenically induced for fetal or maternal reasons, mode of delivery and indications for c-section were recorded. We defined Combined Adverse Maternal Outcome (CAMO) as post-partum hemorrhage (subjective blood loss > 1.000 ml), need of hysterectomy or transfusion of concentrated red cells. Long-term hospital stay was defined as 7 days or more at any time during pregnancy or after delivery.

The main adverse neonatal outcomes included were low Apgar score (< 7 at 5 min), low birth weight (LBW) (< 1500 g and < 2500 g), small for gestational age (SGA) (below the 10th percentile according to local neonatal references), admission to the neonatal intensive care unit (NICU) and neonatal mortality.

PE was defined according to International Society for the Study of Hypertension in Pregnancy (ISSHP) criteria [19]. Gestational hypertension (Systolic Blood Pressure ≥ 140 mmHg and/or Diastolic Blood Pressure ≥ 90 mmHg without significant proteinuria) was a separate outcome that we did not analysed. Late-onset PE was defined as confirmed PE ≥34 weeks.

The diagnosis of gestational diabetes was based on National Diabetes Data Group (NDDG) criteria [20]. According to the protocol, in the presence of polyhydramnios, suspected macrosomia or repeated glycosuria, oral glucose tolerance test (OGTT) was also performed despite having a previous normal O’Sullivan test.

Stillbirth was defined as fetal death occurring after 20 weeks’ gestation or when birth weight was above 400 g.

Placenta previa was diagnosed by transvaginal ultrasound when the placental edge overlapped or was within 2 cm of the internal cervical orifice in late pregnancy.

PPROM was defined as the rupture of amniotic membranes prior to 37.0 weeks of gestation, and was confirmed by the detection of insulin-like growth factor binding protein-1 (Actim PROM test®) in the vaginal fluid. Chorioamnionitis was diagnosed using Gibbs criteria defined in 1982 [21].

Obesity was considered a BMI ≥30. Multiparity was considered when gravida ≥2.

We considered as confounding factors: chronic hypertension, pregestational diabetes mellitus type I/II, use of ART, obesity, smoking, previous c-section and multiparity. Concurrent PE and GD were considered mediating but not contributing factors, so were not included in the multivariate logistic regression analysis.

Permission to conduct the study was obtained from the Institutional Review Board. Number 14/2017719/01.

### Statistical analysis

Mean ± standard deviation was reported for continuous variables, and number and percentage were reported for categorical variables.

A generalized additive model was used to observe the functional distribution of the maternal outcomes over maternal age. According to his relation maternal age was divided into four groups: < 30 years, 30–34 years, 35–39 years, 40–44 years and ≥ 45 years.

Multivariable logistic models were fitted after adjusting for confounding variables and after analyzing the distribution of each outcome.

Odds ratios with their respective confidence interval were calculated for the four interval maternal age groups, using the group aged less than 30 years as a reference. A variable was considered statistically significant when the *p* value was less than α = 0.05.

However, as the sample size was large, the statistical analysis was powerful enough to detect small differences in risk between age groups that might not be clinically relevant. Then, since the OR describes the magnitude of the effect between groups, an aOR ≥ 2 was chosen to represent meaningful risks associated with maternal age. This threshold (OR ≥ 2) was decided on the basis that we consider that the specific management of these patients, including for example additional surveillance, induction of labor or specific prenatal counseling [22], should be adjusted to the increased risk that affects this subgroup. This strategy has been previously used in similar studies with large sample size to stratify risks [7].

The statistical analysis was performed using IBM_SPSS_Statistics V22.0 software. R software version 3.3.2 (2016-10-31) was used for these models [23, 24]. The MGCV statistical software package (https://cran.r-project.org/web/packages/mgcv), ggplot2 (https://cran.r-project.org/web/packages/ggplot2), viridis (https://CRAN.R-project.org/package=viridis) and ggridges (https://CRAN.R-project.org/package=ggridges) were used.

## Results

During the study period, 27723 deliveries were attended in our center. Of those, the following were excluded from the analysis: 2207 (8.0%) multiple pregnancies; 457 (1.6%) pregnancies with confirmed aneuploidies/microdeletions or pregnancy terminations for major defects; and 5 (0.02%) patients under 18 years.

**Fig. 1:**
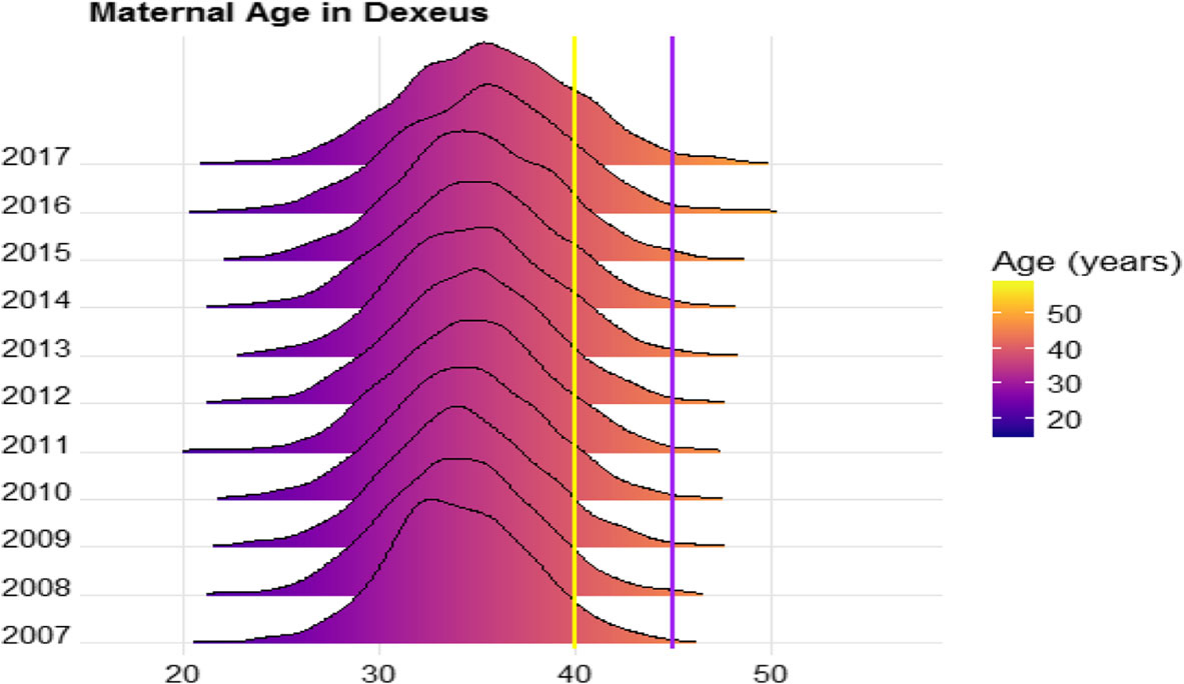
Evolution in the last 10 years of the obstetric population regarding age. Yellow line: 40 years mark. Purple line: 45 years mark

A total of 25054 euploid singleton pregnancies were finally included in the analysis. Mean maternal age was 34.7 ± 4.2 years. In this cohort, 9.7% (2437/25054) of women were <  30 years, 38.5% (9643/25054) were in the range 30–34 years, 39.5% (9887/25054) were 35–39 years, 11.2% (2807/25054) were 40–44 years and 1.1% (280/25054) of patients were ≥ 45 years. Pregnancies in women above 40 years accounted for 7.8% of all pregnant women in 2007 and this prevalence was more than double (19.8%) in June 2017. This increase was even more remarkable among women aged ≥45 years: with a 3.7 fold increase in the last 10 years and accounting for 2.6% of all pregnancies in 2017 (Fig. 1).

Mean global BMI was 23.1 ± 3.7 kg/m^2^: 78.7% of pregnant women had a normal weight (BMI < 25 kg/m^2^), 15.9% were overweight (BMI 25–29 kg/m^2^) and 5.4% obese (BMI ≥30 kg/m^2^).

97.7% of the total population was Caucasian, 99.8% of them from the mediterranean area (Portugal, Italy, Spain, Andorra and Greece). Regarding origin, our population had less migrant patients: 2.3% compared to 32% in the public maternity hospitals of the area. Although information on socio-economic status could not be registered (see limitations), a probably higher socio-economic status (middle-income) than in public hospitals was assumed, as we are a private/mutual health centre.

Summary of maternal characteristics can be seen in Table 1.

**Table 1:** Maternal characteristics per groups of age

	< 30 years *n* = 2437	30–34 years *n* = 9643	35–39 years *n* = 9887	40–44 years *n* = 2807	≥45 years *n* = 280	p
GA at delivery	39.5 ± 1.7	39.5 ± 1.7	39.4 ± 1.7	39.2 ± 1.7	38.6 ± 1.7	< 0,001
Multiparous	7.4%(181)	17.1%(1647)	26.8%(2652)	27.5%(771)	9.3% (26)	< 0,001
Previous c-section	1.9%(46)	4.4%(423)	7.2%(707)	8.8%(247)	6.1% (17)	< 0,001
Chronic hypertension	0.4% (10)	0.4% (38)	0.4% (37)	0.8% (22)	3.6% (10)	< 0,001
Pregestational diabetes	0.6% (15)	0.7%(65)	0.6%(63)	1.0% (29)	–	0,115
Thrombophilias	0.5% (12)	0.6%(61)	1.3%(126)	1.7%(48)	2.9% (8)	< 0,001
ART	1.5% (36)	3.5%(341)	6.9%(683)	17.2%(484)	77.5%(217)	< 0,001
Smoking	13.7%(285)	9.6%(828)	8.4%(756)	9.5%(238)	8.6% (22)	< 0,001
Obesity	6.0%(147)	4.3%(418)	4.7%(468)	5.9%(167)	6.4% (18)	< 0,001

Comparison between maternal characteristics per groups of age. Results are shown as mean ± SD for continuous variables, and % (n) for categorical variables. *P* value was obtained with Chi-square test for categorical variables and T-test for continous variables. *P* < 0.05 was considered significant. *N* number of. *GA* gestational age at delivery. *ART* assited reproductive techniques. *P p* value

### Obstetrical outcomes

**Fig. 2:**
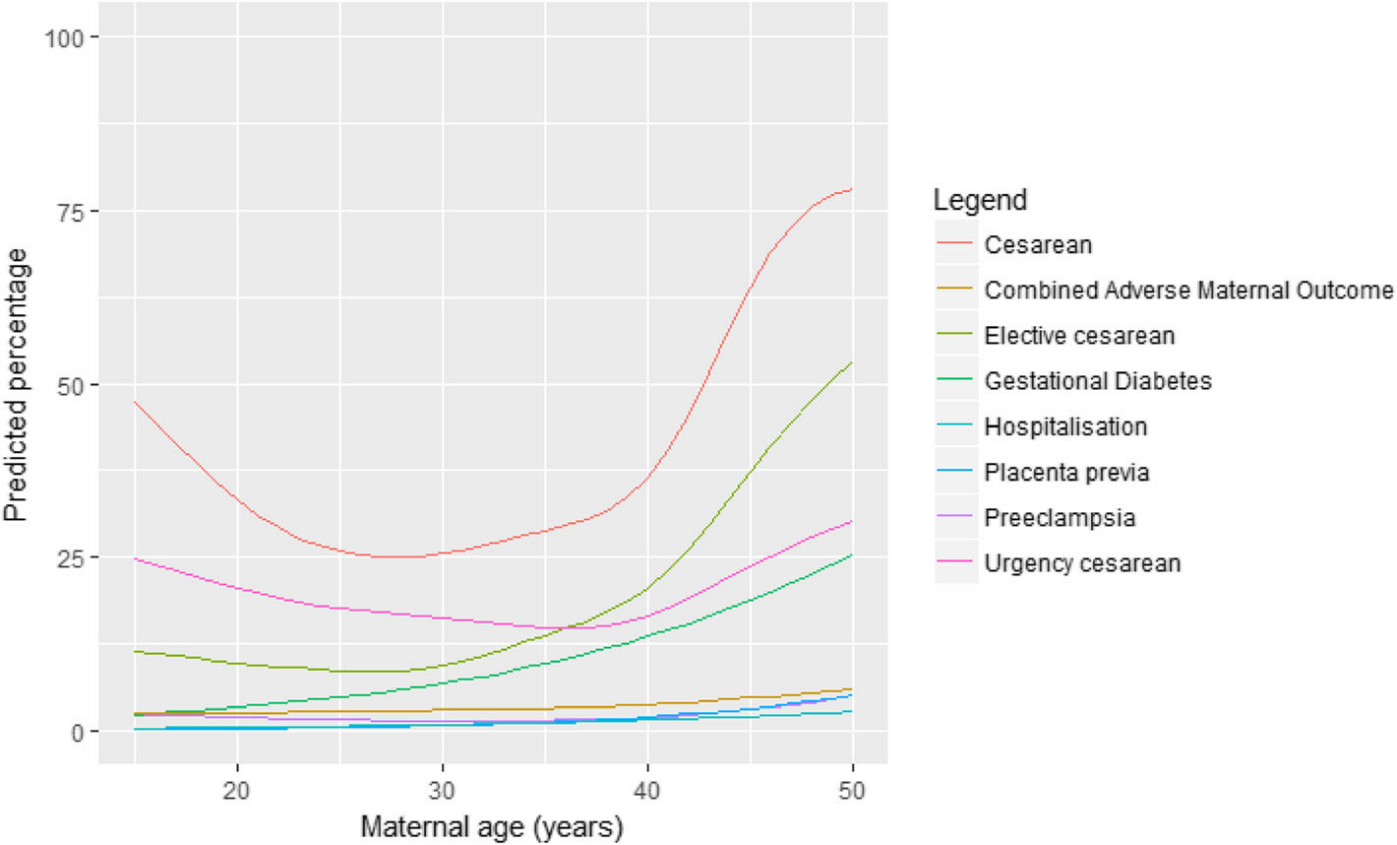
Predictive percentage of main obstetric outcomes (analysing age as a continous variable)

The relation between the obstetrical outcomes and maternal age is shown in Fig. 2. C-section, emergency c-section and elective c-section did not follow a linear distribution but increase or decrease over the age. Maternal age was analysed as a categorical variable by groups of age as specified above (see Statistical analysis).

Age revealed to be statisticallly significantly associated to gestational diabetes and elective c-section in all groups of age compared to the reference group (< 30 years of age). Risk of placenta previa was increased in patients ≥40 years. Preeclampsia, late-onset PE, prolongued hospitalisation and PPROM showed an association only in women ≥45 years. Complete data regarding obstetrical outcomes per groups of age is summarized in Table 2.

**Table 2:** Univariable logistic regression analysis of obstetric outcomes for the different age groups

OBSTETRIC OUTCOMES	Total *n* = 25054	< 30 years -Reference group- *n* = 2437	30–34 years *n* = 9643		35–39 years *n* = 9887		40–44 years *n* = 2807		≥45 years *n* = 280	
	%(n)	%(n)	%(n)	(OR 95% CI)	%(n)	(OR 95% CI)	%(n)	(OR 95% CI)	%(n)	(OR 95% CI)
GD	9.8%(2450)	5.9%(143)	7.9%(758)	1.37 (1.14–1.65)	10.8%(1064)	1.94 (1.62–2.32)	15.4%(431)	2.91 (2.39–3.55)	19.3%(54)	3.83 (2.72–5.39)
PE	1.6%(403)	1.6% (38)	1.5%(140)	0.93 (0.65–1.34)	1.6%(157)	1.02 (0.71–1.46)	1.9%(54)	1.24 (0.82–1.88)	5.0% (14)	3.32 (1.78–6.21)
Late-onset PE	1.2%(309)	1.2% (29)	1.2%(114)	0.99 (0.66–1.50)	1.2%(117)	0.99 (0.66–1.50)	1.5% (41)	1.23 (0.76–1.98)	2.9% (8)	2.44 (1.11–5.40)
Placenta previa	1.3%(321)	0.7% (17)	1.1%(103)	1.54 (0.92–2.57)	1.2%(119)	1.73 (1.04–2.89)	2.3%(65)	3.38 (1.97–5.77)	6.1% (17)	9.20 (4.64–18.24)
C-section	30.4%(7615)	25.2%(615)	26.9%(2597)	1.09 (0.98–1.21)	30.5%(3020)	1.30 (1.18–1.44)	42.1%(1182)	2.16 (1.92–2.43)	71.8%(201)	7.54 (5.72–9.93)
- Emergency C-S	15.9%(3987)	16.4%(400)	15.6%(1509)	0.95 (0.84–1.07)	15.1%(1493)	0.91 (0.80–1.02)	18.1%(508)	1.13 (0.97–1.23)	27.5%(77)	1.93 (1.46–2.56)
- Elective C-S	14.5%(3628)	8.8%(215)	11.3%(1088)	1.31 (1.13–1.53)	15.4%(1527)	1.89 (1.62–2.19)	24.0%(674)	3.27 (2.77–3.85)	44.3%(124)	8.22 (6.25–10.81)
Composite adverse maternal outcome	3.3%(819)	3.2%(77)	3.0%(288)	0.94 (0.73–1.22)	3.3%(328)	1.05 (0.82–1.35)	4.0%(112)	1.27 (0.95–1.71)	5.0% (14)	1.61 (0.90–2.89)
Prolongued hospitalisation	1.0%(247)	0.7% (17)	0.9%(84)	1.23 (0.73–2.07)	1.1%(110)	1.62 (0.97–2.70)	1.1% (30)	1.63 (0.89–2.96)	2.1% (6)	3.69 (1.44–9.46)
Intrahepatic cholestasis of pregnancy	0.8%(189)	0.7% (16)	0.6%(62)	0.98 (0.56–1.70)	0.8%(82)	1.27 (0.74–2.17)	1.0% (27)	1.47 (0.79–2.73)	0.7% (2)	1.09 (0.25–4.76)
PPROM	1.1%(266)	0.9% (21)	1.0%(93)	1.12 (0.70–1.80)	1.1%(106)	1.25 (0.78–1.99)	1.4% (38)	1.58 (0.92–2.70)	2.9% (8)	3.38 (1.49–7.71)
Abruptio placentae	0.3%(67)	0.2% (5)	0.3% (27)	1.37 (0.53–3.55)	0.3% (26)	1.28 (0.49–3.34)	0.3% (9)	1.57 (0.52–4.68)	–	–
Chorioamnionitis	0.1% (29)	0.2% (4)	0.1% (11)	0.70 (0.22–2.18)	0.1% (13)	0.80 (0.26–2.46)	0.03% (1)	0.22 (0.24–1.94)	–	–
PERINATAL OUTCOMES										
Stillbirth	0.2%(49)	0.3% (8)	0.2% (17)	0.54 (0.23–1.24)	0.2% (18)	0.55 (0.24–1.28)	0.2% (5)	0.54 (0.18–1.66)	0.4% (1)	1.09 (0.14–8.73)

*n* number of. *OR* odds ratio. *CI* confidence interval. *GD* gestational diabetes. *PE* preeclampsia. *PP* placenta previa. *C-S* caesarian section. *PPROM* preterm prelabor rupture of membranes

Only one case of maternal death was reported. It happened in a 42 years-old patient, after sudden cardiac arrest during elective c-section.

When adjusting for confounding factors, maternal age still remained an independent risk factor for GD, PP and c-section, but not for PE (neither early nor late-onset PE) (Table 3).

**Table 3:** Multivariable logistic regression analysis of obstetric outcomes and age for main confounding factors (obesity, use of ART, smoking, chronic hypertension, previous c-section and parity)

	GD (aOR 95% CI)	PE (aOR 95% CI)	Late-onset PE (aOR 95% CI)	PP (aOR 95% CI)	c-section (aOR 95% CI)	Elective c-section (aOR 95% CI)	Emergency c-section (aOR 95% CI)
< 30 - Ref group -	1	1	1	1	1	1	1
30–34	1.47 (1.21–1.80)	0.91 (0.62–1.34)	1.01 (0.65–1.56)	1.52 (0.86–2.67)	1.20 (1.06–1.33)	1.29 (1.09–1.53)	1.01 (0.95–1.24)
35–39	2.07 (1.70–2.52)	1.03 (0.71–1.51)	1.06 (0.68–1.64)	1.58 (0.90–2.77)	1.49 (1.33–1.66)	1.82 (1.54–2.15)	1.15 (1.01–1.31)
40–44	3.18 (2.56–3.95)	1.12 (0.71–1.77)	1.18 (0.70–1.99)	2.54 (1.40–4.63)	2.46 (2.15–2.82)	3.17 (2.63–3.82)	1.38 (1.18–1.61)
≥45	3.25 (2.21–4.78)	1.75 (0.83–3.68)	1.28 (0.50–3.30)	3.80 (1.70–8.50)	7.80 (5.79–10.50)	8.09 (5.99–10.92)	2.03 (1.50–2.74)

*Ref* reference. *aOR* adjusted odds ratio. *CI* confidence interval. *GD* gestational diabetes controlled for obesity, smoking, parity and use of ART. *PE* preeclampsia, controlled for chronic hypertension, use of ART, smoking, parity and obesity. *PP* placenta previa, controlled for previous caesarea, parity, smoking and use of ART. *C-section* caesarian section, controlled for previous caesarian section, parity, smoking and obesity

### Perinatal outcomes

Data from 25054 newborns were analyzed. 49 stillbirths (0.19%) were registered and no significant association with advanced maternal age (see Table 2) was observed. Gestational age of stillbirths were: 4 cases ≤24 weeks, 9 cases 24 to ≤28 weeks, 4 cases 28 to ≤32 weeks, 12 cases 32 to ≤36 weeks, 19 cases 36 to ≤40 weeks and 1 case at 41 weeks. 77.5% (38/49) of the stillbirths were of unknown cause. Severe preeclampsia was diagnosed in 1 case at 24 weeks of gestation, there was 1 case of uterine rupture, an infectious cause was confirmed in 5 cases and 4 cases had an umbilical cord accident. Stillbirth cases were excluded from further analysis regarding perinatal outcomes.

No significant associations between maternal age and low Apgar Score (< 7 at min 5) (OR 1.05, 95% CI 0.99–1.11), SGA (OR 0.99, 95% CI 0.98–1.00), preterm delivery < 28 weeks (OR 1.03, 95% CI 0.97–1.09), need of NICU admission (OR 1.02, 95% CI 0.99–1.04) or perinatal mortality (OR 1.01, 95% CI 0.97–1.06) were observed.

In women aged 40 years or above, a significant increased risk of preterm delivery below 34 and 37 weeks was observed: OR 1.04 (95% CI 1.01–1.07) and OR 1.02 (95% CI 1.01–1.04), respectively. However, when adjusting for confounding factors: obesity, ART, parity and smoking (see Table 4), this correlation disappeared. The association remained only significant for iatrogenic prematurity < 37 weeks in women ≥45 years (OR 2.62, 95% CI 1.30–5.27), but not for the spontaneous one.

**Table 4:** Multivariable logistic regression analysis of prematurity and age for confounding factors (obesity, use of ART, smoking and parity)

	PTB < 28 weeks %(n) (aOR 95% CI)		PTB < 34 weeks %(n) (aOR 95% CI)		PTB < 37 weeks %(n) (aOR 95% CI)	
	Spontaneous	Iatrogenic PTB	Spontaneous	Iatrogenic PTB	Spontaneous	Iatrogenic PTB
< 30 (n 2437)	0.2% (4)	0.1% (2)	0.6% (14)	0.3% (7)	2.4%(58)	1.4% (35)
	Ref	Ref	Ref	Ref	Ref	Ref
30–34 (n 9643)	0.1% (13)	0.1% (7)	0.6%(54)	0.4% (35)	2.4%(231)	1.4%(138)
	0.55 (0.17–1.78)	1.63 (0.20–13.32)	0.90 (0.46–1.76)	1.54 (0.60–3.98)	1.03 (0.75–1.43)	1.13 (0.74–1.72)
35–39 (n 9887)	0.2% (18)	0.1% (8)	0.6%(57)	0.5%(45)	2.3%(231)	1.6%(163)
	0.76 (0.25–2.42)	1.72 (0.21–14.05)	1.01 (0.51–1.95)	1.69 (0.66–4.36)	0.99 (0.71–1.37)	1.23 (0.81–1.88)
40–44 (n 2807)	0.1% (3)	0.1% (2)	0.4% (11)	0.2% (19)	0.7%(72)	2.3%(65)
	0.44 (0.08–2.49)	1.41 (0.12–16.31)	0.53 (0.20–1.40)	2.42 (0.86–6.80)	0.95 (0.64–1.43)	1.54 (0.95–2.48)
≥45 (n 280)	0.4% (1)	–	0.4% (1)	1.8% (5)	0.7% (2)	6.1% (17)
	2.69 (0.22–33.20)	–	0.56 (0.07–4.72)	3.41 (0.73–15.91)	0.30 (0.07–1.25)	2.62 (1.30–5.27)

*aOR* adjusted odds ratio. *CI* confidence interval. *N* number of. *Ref* reference group. *PTB* preterm birth

An increased incidence of low birth weight (LBW) < 2500 g (OR 1.01 95% CI 1.00–1.03) and very low birth weight < 1500 g (OR 1.04, 95% CI 1.01–1.08) was observed with advanced maternal age. Nevertheless, maternal age showed up as a non-significant contributor for delivering a LBW baby after adjusting for confounding factors (obesity, parity, use of ART and smoking). Summarized data per age groups can be seen in Table 5.

**Table 5:** Multivariable logistic regression analysis of VLBW and LBW with age for confounding factors (obesity, use of ART, smoking)

	n	VLBW < 1500 (aOR 95% CI)	n	LBW < 2500 (aOR 95% CI)
Total (Inc)		159 (0.6%)		1311 (5.2%)
< 30	15	1	129	1
30–34	51	0.97 (0.48–1.94)	475	0.98 (0.78–1.22)
35–39	70	1.27 (0.64–2.50)	524	1.04 (0.83–1.30)
40–44	20	1.24 (0.56–2.78)	161	1.03 (0.78–1.34)
≥ 45	3	1.81 (0.45–7.23)	22	1.08 (0.64–1.83)

*aOR* adjusted odds ratio. *CI* confidence interval. *Inc.* incidence. *n* number of newborns. *Ref* reference group. *VLBW* very low birth weight. *LBW* low birth weight

## Discussion

In our cohort, 12.3% (3087/25054) of deliveries were from women ≥40 years, in concordance with the social tendency to postpone motherhood in the Spanish population [1, 3].

This study provides evidence that advanced age, after adjustment for main confounding factors (maternal characteristics, smoking, use of ART and main pregnancy complications), is significantly associated to gestational diabetes, placenta previa and caesarean delivery, but not to preeclampsia. No associations with stillbirth, intrahepatic cholestasis of pregnancy, chorioamnionitis and placental abruption were detected.

The risk of these adverse outcomes increased continoulsy with age. Statistical analysis with categorical age groups was conducted to analyze variables with a non-linear tendency and to better compare our research with other previous studies.

The risk of gestational diabetes was positively and significantly related with maternal age even after adjustment for main confounding factors (obesity, smoking, parity and use of ART) specially in women ≥40 years. These results are consistent with most of the previous research assessing this issue [7, 9, 15, 25–27]. The described association between aging and endothelial damage [28, 29] may be the cause: age is a well known cardiovascular risk factor that produces structural and functional changes in the vasculature. Disfunctional endothelium increases the risk of developing insulin resistance [30], which in its turn elevates the risk of hypertension, type 2 diabetes and other metabolic syndromes.

Placenta previa is an uncommon complication that occurs in approximately 0.5% of pregnancies [31]. In this cohort, the incidence in women between 40 and 44 years and ≥ 45 years raised up to 2.3% (65/2807) and 6.1% (17/280), respectively. Even after adjustment for confounding factors, such as previous c-section, parity, smoking and use of ART, these differences remained significant.In the FATER trial, *Cleary-Goldman et al* [7] found a similar risk of placenta previa (AdjOR 2.8 at > 40 years) in a prospective study among more than 36000 patients. This association has also been described in a recent meta-analysis [32], although a main limitation was the lack of adjustment for confounding factors.

Preeclampsia showed a trend for a positive association with age, with an incidence around 1.5% until 40 years and reaching 5% incidence in women ≥45 years. However, no significant association was found at any group of age when adjusting for confounding factors (chronic hypertension, parity, smoking, use of ART, and obesity), neither for early-onset nor for late-onset PE. There is wide evidence on the physiopathology of PE and the higher participation of a maternal impaired endothelial component in late PE [28, 29]. This component would worse with advanced maternal age [30], as age is known to induce endothelial damage that would lead to an increased hypertension risk. Previous research has been controversial when assessing the risk of preeclampsia at an advanced maternal age. Duckitt et al. [33] concluded that women aged ≥40 had twice the risk of developing PE, but all but one study included in the review failed to control preexisting conditions. Lamminpää et al. [12] found that women > 35 years were 1.5-times more likely to have preeclampsia compared to women < 35 years. However, the incidence of preeclampsia in this study was significantly higher (9.4 and 6.4%, respectively) than the one in our population (1.6%). On the contrary, *Cleary-Goldman et al* [7] did not find an association between maternal age and the prevalence of PE, after controlling for parity, history of medical conditions and use of assisted reproductive care. The low incidence of PE in their population (2.4%) and the fact that the same confounding factors were controlled could explain the similarities with our results.

The prevalence of stillbirth in our population was 0.19% (49/25054) and, in line with other previous studies [9, 17], no significant association with age was detected. However, in a recent meta-analysis*, Lean SC et al* [4] concluded that stillbirth risk increases with increasing maternal age. In part, this difference could be explained by the non-distinction between causes of stillbirth in most of the investigations, thus including fetuses with aneuploidies and malformations that were excluded in our analysis. It is noteworthy to mention that in our center, and according to protocol, induction was indicated at above 40 weeks of gestation in those patients ≥40 years. In the 35/39 trial [34, 35] elective induction was performed at 39 weeks if women were > 35 years. To avoid one case of stillbirth, a NNT of 562 was obtained. We could suppose that by raising AMA ≥40 years, the NNT could be somehow lower, although RCT should be addressed to evaluate this issue.

Placental dysfunction disorders such as stillbirth, PE and IUGR were not associated with AMA in our research. The baseline characteristics of our population with a low incidence of PE (1.6%), low obesity rates (5.4%) and our policy of induction at 40 + 0 weeks in women above 40 years of age, could explain these controversial results in comparison with other studies [2, 7, 9, 12, 36].

Overall, 30.4% (7615/25054) of patients underwent caesarean section, a high rate that must be framed in the context of a tertiary referral hospital. After adjusting for confounding factors, such as previous caesarean section, parity and obesity, the prevalence and consequently the risk of c-section significantly increased with maternal age. The same trend was observed regarding elective caesarean section, which was significantly high in pregnant women above 40 years, with a 3-fold increased risk in women between 40 and 44 years and an 8-fold increased risk in women ≥45 years. On the contrary, the risk of an emergency caesarean section only increased significantly among women ≥45 years (AdjOR 2.03, CI 95% 1.50–2.74). Thus, in our population, women < 45 years who had an attempt of vaginal birth did not experience a significant increased risk of emergency c-section. Several previous studies concluded that AMA is associated with higher rates of caesarean delivery [4, 12, 37–40]. This could be explained by a deficient myometrium contractility, atherosclerosis of uterine arteries and a decrease in oxytocin receptors with age [13, 35, 41], However, it remains still controversial whether these higher rates of caesarean delivery, at the expenso of elective c-sections, could only be justified by obstetrical complications and labor dystocia or because patient’s and medical sensitivity towards older pregnancies could lead to iatrogenic interventions [7].

Women ≥45 years showed a significant prolonged hospitalization (> 7 days) in line with the results of C. Haslingera et al. [25] and A. Ben-David et al. [26].

Maternal age was not associated with preterm delivery at any gestational age except women ≥45 years who showed a 2.5-fold increased risk of iatrogenic PTB < 37 weeks. Khalil et al. [9] found a positive association with iatrogenic prematurity when differentiating between both groups of prematurity, but did not control for other confounding factors as was done in our investigation.

A cut-off to define AMA is found to be useful in clinical practice. Women tagged as “AMA” are tributary of greater surveillance and special effort should be made in those patients to prevent complications. In our population, some obstetric risks such as GD and c-section in women between 35 and 39 years were higher than in the reference group but not clinically relevant enough (considering relevant an OR ≥ 2 for the variables studied). Moreover, important outcomes such as placenta previa showed no association with this group of age. In women ≥40 years, significantly higher risks were observed, especially regarding GD (aOR 2.91), PP (aOR 2.56) and c-section (aOR 2.45). Special attention should be focused on women ≥45 years, as it is the group with the highest risks: 19.3% incidence of GD, 6.1% of placenta previa, 71.8% of c-section (including higher risk of emergency c-section), 2.1% of prolonged maternal hospitalization, 6.1% of iatrogenic prematurity < 37 weeks (AdjOR 2.62) and 2.9% of PPROM (OR 3.38). In our population, a definition of AMA above 35 years could dilute obstetric risks and medical efforts on surveillance, and a definition above 45 years could miss many patients at increased risk of relevant adverse obstetric outcomes. Thus, with the information obtained in this study, we propose a cut-off to define AMA ≥40 years for our population.

### Strengths and limitations

Some strengths of this study were: the large number of patients included, fully-controlled in a single center; careful prospective data collection; and detailed information on maternal and neonatal outcomes of interest. This reliable database allowed consistent and accurate retrospective analysis, although a selection bias could not be excluded as it is a single center study. Limitations of this investigation were lack of collected information regarding the patients’ socioeconomic status, impossibility to obtain data on previous preterm birth for multivariable logistic regression analysis in prematurity and a possible selection bias as it is a private/mutual health hospital.

## Conclusions

Maternal age was associated to relevant adverse obstetric outcomes, such as gestational diabetes, placenta previa, caesarean delivery, prolonged hospitalization and PPROM. Although maternal age was significantly and positively associated to these complications in a continuous tendency, women ≥40 years and especially ≥45 years were the ones with higher clinically relevant risks. Moreover, these patients are at increased risk of iatrogenia, as they have very high elective c-section rates, difficult to explain only for obstetrical reasons. Thus, according to these results, a definition of AMA ≥40 years revealed to be adequate in our population. Preconceptional assessment and optimization of previous medical conditions would be recommended before pregnancy in these patients. This study found acceptable good perinatal results, as no significant increase of perinatal mortality or need of NICU admission were observed.

Future investigation should be addressed to develop medical interventions and additional pregnancy surveillance, specially preventive strategies, that could improve pregnancy outcomes in patients with advanced maternal age.

## Data Availability

All data generated or analysed during this study are included in this published article. Supplementary information files are also available on request.

## Abbreviations

AMA: Advanced maternal age
Array-CGH: Array comparative genomic hybridization
ART: Assisted reproductive techniques
BMI: Body mass index
CRL: Crown rump length
EAMA: Extremely advanced maternal age
GD: Gestational diabetes
ISSHP criteria: International Society for the Study of Hypertension in Pregnancy criteria
IUGR: Intrauterine growth restriction
LBW: Low birth weight
NDDG criteria: National Diabetes Data Group criteria
NICU: Neonatal intensive care unit
OGTT: Oral glucose tolerance test
PE: Preeclampsia
PP: Placenta previa
PPROM: Preterm prelabor rupture of membranes
PTB: Preterm birth
SGA: Small for gestational age
VAMA: Very advanced maternal age
VLBW: Very low birth weight
